# Effect of dietary supplementation with yeast cell wall extracts on performance and gut response in broiler chickens

**DOI:** 10.1186/s40104-020-00448-z

**Published:** 2020-05-01

**Authors:** A. Pascual, M. Pauletto, M. Giantin, G. Radaelli, C. Ballarin, M. Birolo, C. Zomeño, M. Dacasto, M. Bortoletti, M. Vascellari, G. Xiccato, A. Trocino

**Affiliations:** 1 Department of Comparative Biomedicine and Food Science (BCA), University of Padova, Viale dell’Università 16, I-35020 Legnaro, Padova Italy; 2 Department of Agronomy, Food, Natural Resources, Animal, and Environment (DAFNAE), University of Padova, Viale dell’Università 16, I-35020 Legnaro, Padova Italy; 3 Histopathology Department, Istituto Zooprofilattico Sperimentale delle Venezie, Viale dell’Università 10, I-35020 Legnaro, Padova Italy

**Keywords:** Gut morphometry, Immuno-histochemistry, *Saccharomyces cerevisiae*, Transcriptomic analysis

## Abstract

**Background:**

The dietary supplementation of yeast cell wall extracts (YCW) has been found to reduce pathogenic bacteria load, promote immunoglobulin production, prevent diseases by pro-inflammatory responses, and alter gut microbiota composition. This study evaluated growth and slaughter results, health, gut morphology, immune status and gut transcriptome of 576 male chickens fed two diets, i.e. C (control) or Y (with 250–500 g/t of YCW fractions according to the growth period). At 21 and 42 d the jejunum of 12 chickens per diet were sampled and stained with hematoxylin/eosin for morphometric evaluation, with Alcian-PAS for goblet cells, and antibodies against CD3+ intraepithelial T-cells and CD45+ intraepithelial leukocytes. The jejunum sampled at 42 d were also used for whole-transcriptome profiling.

**Results:**

Dietary YCW supplementation did not affect final live weight, whereas it decreased feed intake (114 to 111 g/d; *P ≤* 0.10) and improved feed conversion (1.74 to 1.70; *P* ≤ 0.01). Regarding the gut, YCW supplementation tended to increase villi height (*P* = 0.07); it also increased the number of goblet cells and reduced the density of CD45+ cells compared to diet C (*P* < 0.001). In the gut transcriptome, four genes were expressed more in broilers fed diet Y compared to diet C, i.e. cytochrome P450, family 2, subfamily C, polypeptide 23b (*CYP2C23B*), tetratricopeptide repeat domain 9 (*TTC9*), basic helix-loop-helix family member e41 (*BHLHE41*), and the metalloreductase *STEAP4*. Only one gene set (HES_PATHWAY) was significantly enriched among the transcripts more expressed in broilers fed diet Y. However, a total of 41 gene sets were significantly over-represented among genes up-regulated in control broilers. Notably, several enriched gene sets are implicated in immune functions and related to NF-κB signaling, apoptosis, and interferon signals.

**Conclusions:**

The dietary YCW supplementation improved broiler growth performance, increased gut glycoconjugate secretion and reduced the inflammatory status together with differences in the gut transcriptome, which can be considered useful to improve animal welfare and health under the challenging conditions of intensive rearing systems in broiler chickens.

## Background

In a context moving towards antibiotic-free livestock production, research in feed additives has largely increased to evaluate different products and their effects on animal health and immune response, in addition to animal productivity [1]. Among the different products, prebiotics such as yeast cell wall (YCW) extracts have been widely used; they comprise mannan-oligosaccharides (MOS), mannan-proteins, β (1,3)-glucans, β (1,6)-glucans, chitin, and glycophospholipid surface proteins associated with the plasma membrane [2]. The different yeast fractions can modulate animal gut health by different mechanisms.

Dietary supplementation with MOS has been found to improve gut morphology in terms of longer villi, shorter crypts, and a higher number of goblet cells [3]. In infected chicks (*Salmonella typhimurium* and *Clostridium perfringens*), MOS positively affected immune and metabolic pathways in the gut and reduced its colonization [1, 4, 5].

Dietary supplementation with β-glucans increased villi height in the gut and antibody levels against *C. perfringens* [1]. In chicks challenged with necrotic enteritis, β-glucans supplementation also increased the gut expression of genes involved in innate and adaptative immunity, such as cathelicidins and β-defensins [6], while downregulating mRNAs encoding for tight junction proteins (claudin-1, claudin-4, and occludin) in the jejunum [7]. Notably, tight junction proteins are involved in the animal’s protection against infections.

Thus, in broiler chickens, dietary supplementation with YCW extracts containing different rates of MOS and β-glucans might improve growth performance and gut morphology [8–10]; promote the development of immune organs; stimulate gut immunoglobulin secretion; and prevent the colonization of pathogenic bacteria [11, 12]. It can also affect the gut immune and metabolic pathways as measured by means of chicken-specific peptide arrays [5]. In broilers exposed to an experimental necrotic enteritis, YCW supplementation prevented diseases by pro-inflammatory responses (i.e. reduced serum interleukin-1 concentration and increased immunoglobulins G and M) and modified gut microbiota composition through competitive exclusion, production of antimicrobial agents, and change of the fermentation pattern (i.e. increased formic acid and butyric acid levels) in the gut microflora [2].

Nevertheless, to our knowledge, comprehensive data about the effects of dietary supplementation with YCW extracts on the response of broiler chickens in terms of gut inflammatory pattern (CD3+, CD45+), gut whole-transcriptome profiling, animal performance, and gut morphology are still lacking. Therefore, the present study aimed at evaluating the effect of dietary supplementation with YCW extracts (mainly mannan-oligosaccharides and β-glucans) from *Saccharomyces cerevisiae* (SafMannan®, Phileo, Lesaffre, Marcq-en-Baroule Cedex, France) on growth performance and slaughter results, health, gut morphology, immune status and gut transcriptome in broiler chickens.

## Methods

The trial was performed at the poultry house of the Experimental Farm of the University of Padova (Legnaro, Padova, Italy), after a long period of downtime (about 6 months). The study was approved by the Ethical Committee for Animal Experimentation of the University of Padova. All animals were handled according to the principles stated by the EC Directive 86/609/EEC [13] about the protection of animals used for experimental and other scientific purposes.

### Experimental facilities

The poultry house was equipped with a cooling system, forced ventilation, radiant heating and controlled light system. A total of 24 wire-net pens (3.0 m^2^; 120 cm wide × 250 cm large × 120 cm height) were used, each equipped with 5 automatic nipple drinkers and a circular feeder (diameter: 37 cm) for manual distribution of feed. The pens had a concrete floor bedded by wood shavings-wheat straw litter (height 5 cm, 2.5 kg/m^2^). Twenty-four hours of light were provided during the first 2 d after the chickens arrived at the poultry house. After the first 2 d, hours of lights were progressively reduced until a 18L:6D light program was reached, which was maintained from the 13^th^ day onwards.

### Animals, experimental groups and *in vivo* recordings

A total of 576 male chickens, commercial crossbred Ross 308 (Aviagen Group, USA) were transported by appropriate and authorized transport means to the experimental facilities of the University on the hatching day. All chicks had been vaccinated against Marek’s disease, Infectious Bronchitis (H120 + 793B) and Newcastle disease at the hatchery. On their arrival, 24 chicks per pen were placed in 24 pens, randomly allocated to two experimental groups (12 pens per group), i.e. two dietary treatments: C, control, and Y, supplemented with YCW extracts (Safmannan®).

Chicks were individually weighed on their arrival, identified by a leg ring, and weighed for live weight once a week until slaughtering at 44 d. Pen feed consumption was measured daily during the trial.

### Diets and feeding plans

For each treatment, three commercial diets in crumble form were administered during the trial as usual, i.e. diet C1 and diet Y1 from 1 to 14 d; diet C2 and diet Y2 from 15 to 28 d; and diet C3 and diet Y3 from 29 d until slaughtering (on d 44) (Table 1). All diets were formulated to satisfy broiler nutritional requirements [14]. Diet C1 contained: corn, soybean meal, full fat soybean, corn gluten, monocalcium phosphate, animal fat, soybean oil, sodium bicarbonate, sodium chloride, besides vitamin-mineral premix, phytase, and coccidiostat. Diet C2 included: corn, soybean meal, animal fat, calcium carbonate, monocalcium phosphate, corn gluten, sodium bicarbonate, sodium chloride, besides vitamin-mineral premix, phytase, and coccidiostat. Diet C3 included: corn, soybean meal, animal fat, corn gluten, monocalcium phosphate, sodium chloride, and vitamin-mineral premix. The diets Y (Y1, Y2, and Y3) contained different levels of Safmannan® (mannan-oligosaccharides > 20%; β-glucans 1,3 and 1,6 > 20%) as source of YCW extracts (250, 500, and 250 mg/kg in the three diets, respectively) as used in the field. All diets were produced by a commercial feed mill (Fanin s.r.l., San Tomio di Malo, Vicenza, Italy). The inclusion of Safmannan® in the diets Y was done at the feed mill at the production time according to current practices.

**Table 1:** Chemical composition of experimental diets

Period of administration	1–14 d		15–28 d		29–44 d	
Diet	Control	Yeast^a^	Control	Yeast^b^	Control	Yeast^c^
Dry matter, g/kg	899	897	887	893	889	894
Crude protein, g/kg DM	231	236	221	214	183	183
Ether extract, g/kg DM	52.6	52.4	70.6	77.0	63.1	65.8
Crude fibre, g/kg DM	17.2	18.0	18.0	20.6	17.5	16.0
Ash, g/kg DM	74.3	71.3	74.7	78.1	62.9	61.5
N-free extracts, g/kg DM	625	623	616	611	674	674

^a^It contains 250 mg/kg of Safmannan® as source of YCW extracts

^b^It contains 500 mg/kg of Safmannan® as source of YCW extracts

^c^It contains 250 mg/kg of Safmannan® as source of YCW extracts

The diets were analysed to determine the contents of dry matter (934.01), ash (967.05), and crude protein (2001.11) by AOAC [15] methods. Ether extract was analysed after acid hydrolysis [16].

### Gut sampling

At both 21 d and 42 d of age, 24 chickens (1 chicken per pen; 775 ± 160 g at 21 d and 2,085 ± 169 g at 42 d) were selected on treatment-wise average live weight and slaughtered by cervical dislocation to sample gut tissue. One sample of about 2 cm was taken from jejunum at the midpoint between the end of the duodenal loop and the location of the Meckel’s diverticulum [17] and washed in phosphate buffer saline (PBS) solution. About 1 cm was fixed in paraformaldehyde in PBS (0.1 mol/L, pH 7.4) to be later dehydrated, and embedded in paraffin at the lab. Exclusively for chickens slaughtered at 42 d of age, just before fixing, small circular sections were also collected in RNase-free conditions, immerged and stored in RNAlater reagent (Applied Biosystems, Foster City, CA, USA) for transcriptomic analyses. Once in the lab, samples for RNA-Seq were stored at 4 °C overnight, and then transferred to − 80 °C until further processing.

### Histological analyses and immunohistochemistry

Two serial sections of 4 μm per sample were obtained using a microtome and stained with haematoxylin/eosin for morphometric evaluation and with Alcian blue (pH 2.5)-PAS method for a quantitative and qualitative analysis of goblet cells, respectively. Moreover, two serial sections were used for CD3+ and CD45+ immunohistochemical analysis for the sampling at 42 d of age. The villi length and the depth of the crypts were measured with image-analysis software (DP-soft, Olympus Optical, Co., Hamburg, Germany) according to the procedure described by Hampson [18], with the aim of collecting an average of 30 measurements from each animal. The goblet cells positive to Alcian blue (pH 2.5)-PAS staining were counted on 10 different villi per animal along 500 μm of each villus surface. Along the same distance, the area of goblet cells was measured by image analysis. For broilers slaughtered at 42 d of age, serial sections were used for immuno-histochemical analyses following the procedure described by Röhe et al. [19] to determine CD3+ intraepithelial T- cells as well as CD45+ intraepithelial leukocytes in the jejunal tissue of broilers. Intraepithelial leukocytes were counted in the epithelium using a reference rectangle with the short side at 100 μm and were expressed as density of CD45+ and CD3+ (cells/10,000 μm^2^).

### RNA-Seq library preparation and sequencing

After RNAlater solution removal, tissues were ground using a steel pestle and total RNA was extracted using the RNAeasy Mini Kit (Qiagen, Hilden, Germany), following the manufacturer’s instructions. The RNA concentration was determined using a NanoDrop ND-1000 spectrophotometer (NanoDrop Technologies, Wilmington, DE, USA), and its quality assessed with a 2100 Bioanalyzer (Agilent Technologies, Waldbronn, German). All samples had an RNA Integrity Number (RIN) value > 7.

Equal amounts of RNA (i.e., 500 ng) of three different chicks from the same experimental group were pooled together, thus obtaining a total of four RNA pools (i.e. replicates) for each of the two dietary treatments. Therefore, RNA-Seq data were representative of a total of 24 animals (12 per group). A total of 8 tagged RNA-Seq libraries were prepared from RNA pools using Agilent’s SureSelect Strand Specific RNA Library Preparation Kit (Agilent Technologies, Waldbronn, German), following the manufacturer’s instructions. Briefly, poly(A) mRNA was purified from 1.5 μg of total extracted RNA and fragmented using an RNA-Seq Fragmentation Mix. First-strand and second-strand cDNA were synthetized and end-repaired. Adenylation of cDNA 3′ ends and adaptor ligation were performed. Twelve cycles of PCR were used to amplify and index the adaptor-ligated cDNA library, and the PCR products were purified and size selected using the SPRIselect reagent kit (Beckman Coulter, Brea, CA, USA). Library concentrations were measured by both a Qubit RNA Assay kit in a Qubit 2.0 Fluorometer (Life Technologies, Carlsbad, CA, USA) and a PCR-based method by using the NEBNext Library Quant Kit for Illumina (New England Biolabs, Ipswich, MA, USA). Individual libraries were monitored for insert size using the Agilent DNA 1000 assay on the Agilent Bioanalyzer 2100 system (Agilent Technologies). Multiplexed single-end sequencing 50 bp was carried out on an Illumina Hi-Seq 4000 (Fasteris, Geneva, Switzerland).

### RNA-Seq reads processing and mapping

Initial quality control was carried out with the FastQC software version 0.11.5 [20]. Reads were trimmed and adapters were removed using Trimmomatic (version 0.36) with default parameters [21]. Reads shorter than 36 bps were excluded. To filter out any remaining post-sequencing ribosomal RNAs (rRNAs), the local sequence alignment tool SortMeRna 2.0 [22] was applied against different databases (Rfam 5.8S; Rfam 5S; Silva 16S archaeal, bacterial; Silva 18S eukaryote; Silva 23S archaeal, bacterial; Silva 28S eukaryote). Reads were then mapped against the reference genome available in Ensembl (GCA_000002315.5) using the STAR aligner and following the two-pass mapping mode [23]. The maximum number of allowed mismatches and the maximum number of loci the read can map to were set to 4 and 10, respectively. Read counts for each sample, at the gene level, were extracted by setting the *GeneCounts* quantification while running STAR.

### Commercial slaughtering

At 44 d of age, all chickens were slaughtered in a commercial slaughterhouse. Feed and water were removed at the same exact time for all chickens, corresponding to approximately 7 h feed and 4 h water withdrawal, respectively, before slaughtering. Chickens were individually weighed before crating. All chickens of a pen were loaded in a transport cage. Chickens were slaughtered according to the standard practice of the commercial slaughterhouse (slaughter line speed: 6,000 chickens/h). Ready-to-cook carcasses were recovered after 2 h of refrigeration at 2 °C.

A total of 120 carcasses (five per pen, 60 per dietary treatment), which had been previously selected on the basis of the slaughter live weight to be representative within a pen, were individually weighed to measure slaughter dressing percentage and transported to the Department laboratories to be stored at 2 °C. Twenty-four hours after slaughter, carcasses were dissected for the main cuts, i.e. breast and hind legs [24].

### Statistical analysis

Individual data related to live weight and daily growth was analysed with a generalized linear mixed model considering the diet as the main factor of variability and the pen as a random effect using PROC MIXED [25]. Cage data related to feed intake and feed conversion were analysed with a generalized linear model using PROC GLM to test the effect of the diet. Individual data related to gut morphology, goblet cell and CD3+ and CD45+ cell density were analysed with the same model, using diet, age (when relevant) and their interactions as main effects. The Chi Square test was used to test differences for mortality according to the diet. Adjusted means were compared by Bonferroni-*t* test. Differences between means with *P ≤* 0.05 were accepted as statistically significant. Differences between means with *P ≤* 0.10 were accepted as representative of a statistically significant trend.

Regarding whole-transcriptome profiling, a pairwise Differential Expression (DE) analysis was performed using Deseq2 (Wald Test adjusted *P ≤* 0.10, log_2_ fold change (log_2_FC) > 1) [26]. Using the output of the DE analysis, a Preranked Gene Set Enrichment Analysis (GSEA) [27] was employed to determine whether gene sets defined a priori show statistically significant enrichment at either end of the ranking. A statistically significant enrichment (nominal *P ≤* 0.05) indicates that the biological activity (e.g., the biomolecular pathway) characterized by the gene set is correlated with the supplied ranking. As GSEA is typically used with gene sets from the Molecular Signatures Database (MSigDB), consisting of HUGO (Human Genome Organization) gene symbols, chicken gene IDs were used to obtain human orthologues and related HUGO gene symbols in BioMart. The input was prepared as follows: the raw *P*-values obtained through the DE analysis (i.e. Deseq2, “diet Y” versus “diet C”) were used to rank the list of genes by significance. When multiple genes with the same gene symbol were detected, only the most significant one (based on *P*-value) was retained. To specify the direction of the gene expression variation, the *P*-values (*P*) were replaced by 1−*P* or −(1−*P*) when a gene was over-expressed or under-expressed in chickens fed diet Y, respectively. The analysis was carried out by using a *classic* enrichment statistic and the MSigDB curated gene sets belonging to the BIOCARTA collection.

## Results

### Productive performance

On the hatching day, the weight of chicks averaged 46.2 ± 3.2 g without significant differences between experimental groups (Table 2). However, at 14 d and 28 d of age, birds fed diet Y were lighter than those fed diet C (− 2.6%, *P* = 0.08; and − 2.9%, *P* = 0.03, respectively). At 44 d of age, the average live weight was 2,964 g without significant differences between the two dietary treatments.

**Table 2:** Growth performance of broiler chickens

	Dietary treatment		*P-*value	RMSE^a^
	Control (C)	Yeast (Y)		
Broilers, *n*	226	229		
Live weight, g				
1 d	45.9	46.5	0.11	3.40
14 d	422	411	0.08	54.1
28 d	1494	1451	0.03	134
44 d	2959	2968	0.83	230
Period 1 (1–14 d)				
Daily weight gain, g/d	28.9	28.0	0.06	4.11
Feed intake^b^, g/d	34.1	33.6	0.23	1.05
Feed conversion index^b^	1.18	1.20	0.10	0.03
Period 2 (14–28 d)				
Daily weight gain g/d	76.6	74.3	0.04	6.78
Feed intake^b^, g/d	109	105	< 0.01	3.00
Feed conversion index^b^	1.43	1.42	0.40	0.02
Period 3 (28–44 d)				
Daily weight gain, g/d	91.6	94.8	0.06	10.5
Feed intake^b^, g/d	183	180	0.34	7.34
Feed conversion index^b^	2.00	1.90	< 0.001	0.05
Whole trial (1–44 d)				
Daily weight gain, g/d	66.2	66.4	0.84	5.22
Feed intake^b^, g/d	114	111	0.09	3.43
Feed conversion index^b^	1.74	1.70	0.01	0.03

^a^RMSE, root mean square error

^b^Pen-based data

During the first period of growth (1–14 d), dietary supplementation with YCW extracts tended to decrease daily weight gain (*P* = 0.06) without effects on feed intake, which tended to impair feed conversion (*P* = 0.10) compared to diet C (Table 2). During the second period (14–28 d), YCW supplementation decreased both daily weight gain (*P* = 0.04) and feed intake (*P* ≤ 0.01), without effects on feed conversion. Conversely, during the third period (28–44 d), the YCW supplementation tended to promote daily weight gain (*P* = 0.06) without a difference in feed intake and, thus, significantly improved feed conversion (*P* ≤ 0.001). In the whole trial, YCW supplementation did not affect daily weight gain, whereas it tended to decrease feed intake (*P* = 0.09) and improved feed conversion (*P* = 0.01) (Table 2).

Total losses due to mortality during the trial ranged from 9.85% to 6.82% in animals fed diets C vs. those receiving diets Y (*P* = 0.21) (data not reported in tables).

### Slaughter results and carcass traits

Slaughter results and carcass quality did not change according to the dietary treatment (Table 3). The weight of carcasses averaged 2,182 g, corresponding to a dressing out percentage of 73.6%. The average breast (with bones) proportion on the whole carcass was equal to 39.4% on average and tended to be lower in chickens fed the control diet compared to those fed the diet Y (39.1% vs. 39.8%; *P* = 0.07), whereas the proportions of other main carcass cuts of carcass did not differ between dietary treatments.

**Table 3:** Carcass traits of broiler chickens at slaughter

	Dietary treatment		*P-*value	RMSE^a^
	Control (C)	Yeast (Y)		
Broilers, *n*	60	60		
Slaughter live weight, g	2980	2977	0.94	196
Carcass weight (CC), g	2187	2177	0.74	157
Dressing out percentage, %	73.7	73.4	0.35	1.56
Breast, % CC	39.1	39.8	0.07	4.69
*P. major,* % CC	24.3	24.7	0.24	1.53
Hind legs, % CC	29.6	29.3	0.37	1.31

^a^RMSE, root mean square error

### Gut morphology and immuno-histochemical analyses

**Fig. 1:**
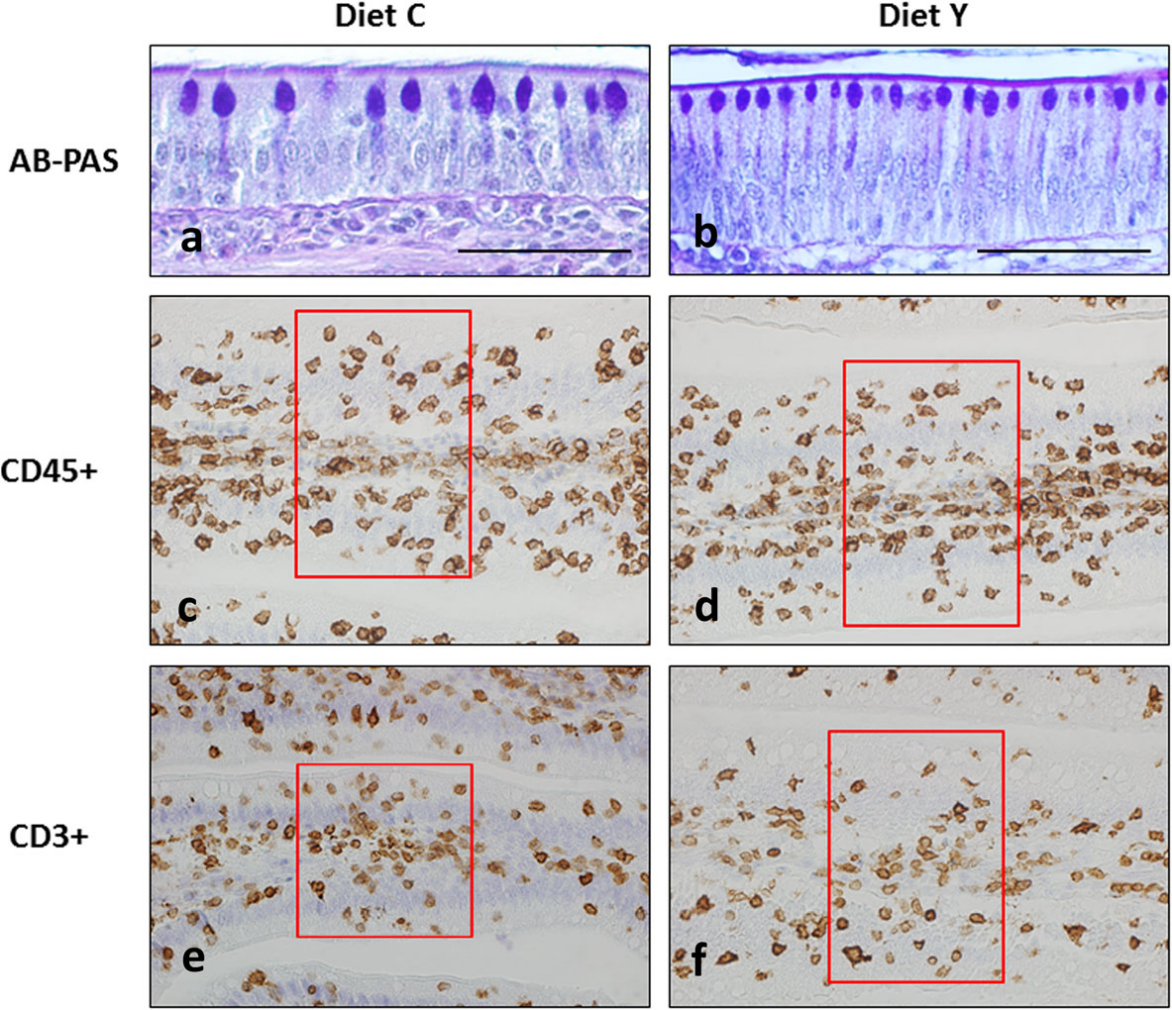
Representative light micrographs of jejunum from chickens at 42 d of age fed the diet C (**a**) or diet Y (**b**) after AB-PAS staining. Bar = 50 μm. Immuno-histochemical staining of jejunum from chickens at 42 d of age fed the diet C (**c, e**) or diet Y (**d, f**) for the determination of cells positive to CD45 (**c, d**) or CD3 (**e, f**). The rectangle delimits the area of the epithelium in which intraepithelial positive cells were counted (short side at 100 μm)

The effect of the dietary treatment on gut morphology approached statistical significance only in the case of the villi height (*P* = 0.07), which was higher in chickens fed diets Y compared to those fed diets C (Table 4). Also, the density of goblet cells was significantly (*P ≤* 0.001) higher (47.0 vs. 21.7 cells/500 μm) and the area of the same cells lower (14.4 vs. 39.2 μm^2^) in chickens fed diet Y compared to chickens fed diet C (Table 4) (Fig. 1a, b). Finally, the density of cells positive to both CD45+ (*P* ≤ 0.001) and, at a lower extent, CD3+ (*P* = 0.08) were significantly lower in birds fed diet Y compared to those fed diet C.

**Table 4:** Gut morphometry, number of goblet cells and density of CD45+ and CD3+ cells at 21 and 42 d of age

Age (A)	21 d		42 d		*P*-value			RMSE^e^
Dietary treatment (D)	Control (C)	Yeast (Y)	Control (C)	Yeast (Y)	A	D	A×D	
Broilers, *n*	12	12	12	12				
Villi height, μm	1011	1086	1350	1363	< 0.001	0.07	0.19	180
Crypt depth, μm	151	162	140	141	0.03	0.12	0.17	31
Villi to crypt ratio	6.70	6.70	9.64	9.67	< 0.001	0.71	0.79	1.27
Goblet cells density, cells/500 μm	24.3^b^	54.3^d^	19.1^a^	39.6^c^	< 0.001	< 0.001	< 0.001	3.61
Goblet cells area, μm^2^	40.5^d^	12.0^a^	37.8^c^	16.7^b^	< 0.001	< 0.001	< 0.001	8.72
CD45+, cells/10,000 μm^2^	–	–	2972	2728	–	< 0.001	–	549
CD3+, cells/10,000 μm^2^	–	–	2180	2037	–	0.08	–	639

^a, b, c, d^Values with different superscript letters significantly differ *(P* < 0.05)

^e^RMSE, root mean square error

Figure 1c and d show gut tissues positive to CD45+. Since a high number of positive cells strictly in contact each other were present in the connective tissue of the lamina propria, only intraepithelium cells were counted and referred to the area size identified by the red rectangle in the figure. The same was true for cells positive to CD3+ shown in Fig. 1e and f.

Regarding the effect of age, villi height significantly increased (1,049 μm to 1,357 μm; *P* ≤ 0.001) and crypt depth decreased (157 μm to 141 μm; *P =* 0.03) from 21 to 42 d. As age increased, the density of goblet cells decreased (39.3 to 29.3 cells/500 μm; *P* ≤ 0.001) (Table 4).

### Whole transcriptome analysis

A total of 194,901,804 raw reads were obtained and deposited in GeneBank. All samples passed quality control measures for raw sequenced reads. After trimming and rRNAs removal, an average of almost 22 million reads per sample were retained, with ~ 96% of reads mapping to the *Gallus gallus* reference genome. Numbers of raw reads, reads passing the filters, and reads mapping reads for each library are provided in Supplementary Table S1, Additional File 1.

The DE analysis showed only slight differences in the gut transcriptome of broilers according to the dietary treatment. A total of 24,145 genes were assessed. Genes whose mRNA levels were significantly different (adjusted *P* ≤ 0.10, log_2_FC > 1) are reported in Table 5. Specifically, four genes were more expressed in broilers fed diet Y compared to those fed diet C, i.e. cytochrome P450, family 2, subfamily C, polypeptide 23b (*CYP2C23B*), tetratricopeptide repeat domain 9 (*TTC9*), basic helix-loop-helix family member e41 (*BHLHE41*), and the metalloreductase *STEAP4*.

**Table 5:** Differentially expressed genes in broiler gut transcriptome at 42 d of age: diet Y

Gene ID	Gene name	Gene description	Log_2_FC^a^	Adjusted *P*-value^b^
ENSGALG00000005795	*CYP2C23b*	Cytochrome P450, family 2, subfamily C, polypeptide 23b	1.21	2.46E-08
ENSGALG00000017652	*TTC9*	Tetratricopeptide repeat domain 9	1.31	0.015
ENSGALG00000048539	*BHLHE41*	Basic helix-loop-helix family member e41	1.26	0.019
ENSGALG00000008997	*STEAP4*	STEAP4 metalloreductase	2.00	0.082

^a^Positive log_2_ fold changes (log_2_FC) stand for an up-regulation in diet Y vs. diet C

^b^Benjamini–Hochberg *P-value* correction

Results from GSEA showed that only one gene set (HES_PATHWAY, i.e. “Segmentation Clock”) was significantly enriched among the genes more expressed in broilers fed diet Y. However, a total of 41 gene sets were significantly over-represented among genes up-regulated in control broilers. Notably, several enriched gene sets are implicated in immune functions and related to NF-κB signaling, apoptosis, and interferon signals (e.g. “Double Stranded RNA Induced Gene Expression”, “NO2-dependent IL 12 Pathway in NK cells”, “NF-κB Signaling Pathway”, “Induction of apoptosis through DR3 and DR4/5 Death Receptors”, “Toll-Like Receptor Pathway”, “Chaperones modulate interferon Signaling Pathway”, “T Cytotoxic Cell Surface Molecules”, “FAS signaling pathway”, and “Signal transduction through IL1R”). The complete list of enriched BIOCARTA gene sets is provided in Supplementary Table S2, Additional File 1.

## Discussion

Among the different feed additives, YCW extracts have been widely used in animal feeding and, specifically, in broiler chickens. Nevertheless, their effects on performance and health are not always consistent among studies, because several conditions may differ. Firstly, the contents of the different YCW fractions (e.g. MOS, β-glucans) can vary in the different products according to fermentation conditions and cultivation methods of the yeast as well as of *S. cerevisiae* groups and their substrates (wine, beer, and bread) [5, 28]. Also different inclusion rates of YCW extracts have been tested in the studies so far published. Moreover, these studies have been conducted following specific experimental conditions (length trial, presence or in absence of specific challenges and stress conditions).

Based on the literature, when no specific challenge was applied, the dietary supplementation with YCW extracts in the range tested in our trial (i.e. 250–500 ppm) did not produce significant improvements in growth performance of broilers in the starter period (21 d) [12, 29]. Contrarily, at higher supplementation levels (1,000–2,000 ppm) and/or on longer growth periods, feed conversion improved, as in the present study, and/or final live weight increased [8, 10, 11, 30]. Using a yeast derivative made by yeast cell wall fragments and yeast extracts, Reisenger et al. [9] recorded the best results with the lowest supplementation rate (1,000 ppm vs. control and 2,000 ppm).

In broilers at 21 d of age previously submitted to an immune stress and a *C. perfringens* challenge, the dietary supplementation tested in the present trial (Safmannan®) had a positive impact on final body weight, weight gain and feed conversion ratio [31]. A positive effect of YCW supplementation on growth performance was also recorded in broilers challenged for New Castle disease [8] and for *Salmonella typhimurium* [4] as well as in the case of chickens fed diets contaminated with aflatoxin B1 and ochratoxin A [32]. YCW supplementation also decreased mortality in broilers challenged with dietary aflatoxin B1 and necrotic enteritis [33].

Under our conditions, the reduction in daily growth observed during the first and, especially, the second period of growth (15 to 28 d of age) might be related to the higher YCW supplementation rate used in this period (i.e. 500 vs. 250 ppm). In fact, Fowler et al. [31] estimated the optimal dose of YCW supplementation (provided as Safmannan or Pronady) is 250 ppm, whereas higher doses could negatively impact broiler performance. The YCW supplementation could have had a trophic effect on the gut rather than on muscle accretion as proved by the increased villi length observed in our trial, especially at earlier ages (21 d vs. 42 d). Literature results about the effects of yeast fractions and YCW extracts on gut morphology are not always consistent and vary according to the yeast fractions and the testing conditions. Some authors reported that either MOS [34] or yeast cell and YCW extracts [9, 35, 36] did not affect broiler gut morphology. According to others, MOS increased villi length in comparison to antibiotic supplementation (virginiamycin and bacitracin) [35] or to a control diet [8, 37]. These changes in gut morphology have been associated with changes in growth performance because of increased absorption surface.

On the other hand, according to Reisinger et al. [9], a high inclusion rate of YCW extracts could decrease animal performance because of a potential over-reaction of the immune system. In fact, under our conditions a higher density of goblet cells was recorded in chickens fed diets supplemented with YCW compared to the control diet, and in chickens at 21 d of age compared to 42 d suggesting that YCW induces a proliferation of goblet cells as a defense mechanism. The higher density of goblet cells at earlier ages could be attributed to the higher YCW inclusion rate fed at the first sampling time compared to the second one. Regarding YCW supplementation, other authors have also reported an increased density of goblet cells in broilers fed diets supplemented with YCW extracts [8, 9, 29, 36].

Interestingly, the increased density of the goblet cells in animals fed diet Y went about with decreased cells size compared to chickens fed the control diet. It is possible that in the control group, cells were larger because they were storing and accumulating mucin and the stimulus to release the mucin was lower, whereas in the case of chickens fed diet Y several cells were continuously producing and releasing mucus [38].

The increase in the number of goblet cells can be positively considered in view of the protective effect of mucus production by goblet cells under any challenging conditions [9], but under our conditions it could have somewhat impaired animal performance during the first period of growth. Later, during the last period, chickens fed diet Y showed a higher growth rate compared to those fed diet C and a better feed conversion. Indeed, the counts of the cells positive to CD45+ and CD3+ showed a better response for the broilers fed diet Y compared to those fed diet C at 42 d of age.

Only a few studies are available on local gut associated immune reactions in poultry and they refer to the effect of different dietary protein sources (soybean vs. differently processed peas) [19]. Our study showed differences in the mucosal immune responses as the density of intraepithelial leukocytes (especially CD45+) was lower when chickens received a diet supplemented with yeast cell walls. A lower immune reaction in these birds could mean that YCW protected animals from any agent/cause that could have challenged animals during the trial, which is consistent with the increased number of goblet cells, and thus increased mucin production, in the gut of these animals [39]. In fact, Röhe et al. [19] found that jejunal intraepithelial T cells increased because of feeding based on legumes most likely because of their contents in antigenic proteins, lectins or other anti-nutritional factors triggering the local gut immune response. In our study, we were not able to assess the inflammation causing agents and, possibly, more factors contributed to the gut status, but dietary YCW may have contributed differently to the reduction of inflammation due to their components (MOS and glucans).

In our trial, further insights in the effects of YCW supplementation on the immune status of animals were obtained by whole transcriptome analysis (i.e. RNA-Seq). Based on the literature, the four genes whose expression was significantly increased by YCW supplementation might play pivotal roles in immune responses, but these traits have not yet been extensively investigated in chickens. Notably, the most significant differentially expressed gene (DEG) was *CYP2C23B*, whose expression was 2.3 fold higher in broilers supplemented with YCW. This drug-metabolizing enzyme (an epoxygenase) catalyzes the conversion of arachidonic acid in epoxyeicosatrienoic acids (EETs), short-lived signaling eicosanoids. Previous studies showed EETs possess anti-inflammatory and antiapoptotic activity in kidney [40] as well as in cardiovascular endothelium by inhibiting NF-κB activation [41]. As far as *TTC9* is concerned, little information about its role in the gastrointestinal tract is available. Nevertheless, we cannot exclude this gene might contribute to chicken gut homeostasis, as the human isoform *TTC7A* is crucial in maintaining intestinal epithelial cell integrity [42]. The transcription factor *BHLHE41* regulates the mammalian molecular clock; interestingly, in humans it controls the B-cells receptor repertoire, the self-renewal of B-1 cells, and innate-like B-lymphocytes, thus providing a first line of defence against pathogens [43]. Finally, STEAP4 (TNFAI9) has been shown to suppress IL-6 production and TNF-α-induced NF-κB signalling in murine macrophages [44]. Thus, it is conceivable that this gene might have anti-inflammatory properties in broiler jejunum too.

The GSEA analysis pointed out additional results elucidating the processes triggered by YCW-supplemented diets in chickens. The pathway mediated by the epithelial transcription factor *HES1* was enriched among the up-regulated genes in birds fed diet Y. This is of particular interest, since *HES1* is a transcription factor proposed to be one of the primary mediators of intestinal Notch signals, which in turn contribute to epithelial cells choice of an absorptive versus a secretory lineage [45]. In particular, *HES1*, through the repression of a secretory lineage transcription factor (i.e. *HATH1*), drives cells to become absorptive enterocytes [46]. Thus, the higher expression of genes belonging to *HES1* pathway in animals fed diet Y seems to be consistent with the observed increase of villi height after yeast-supplementation, giving both these mechanisms a role in nutrient absorption. Nonetheless, in mice *HES1* has been identified as a homeostatic suppressor of inflammatory responses both *in vivo* (i.e. serum) and *in vitro*(i.e. primary macrophages) [47].

Interesting results were also noticed for downregulated genes in chickens fed diet Y. The GSEA analysis pointed out a significant enrichment of several genes sets, most of them being related to viral infection response (e.g. double-stranded RNA activated pathway, interferon signalling pathway), antigen recognition (e.g. activation of T-cell receptors, toll-like receptor pathway, T-cell cytotoxic surface proteins), stress response (e.g. NF-κB activation), apoptosis induction (FAS signalling pathway), and inflammation (signal transduction through interleukin-1 receptor). Overall, this demonstrates that immune, pro-inflammatory and pro-apoptotic processes are activated to a higher extent in chicks fed the control diet compared to those fed with the supplemented diet. The activation of these pathways might be a consequence of higher stress levels due to any challenging condition (e.g. nutritional challenge, pathogens infection, inflammatory processes) in control animals, and is in agreement with the higher density of intraepithelial leukocytes and the reduced amount of goblet cells observed in this experimental group.

Considering the limited number of DEGs, we can reasonably conclude that the yeast-supplemented diet tested in this study induce small changes in the gut transcriptome of broiler chickens. Indeed, results provided by the GSEA, which is powerful in detecting even small but coordinated transcriptional changes, are of particular interest, as they are highly consistent with the hypothesis that yeast supplementation might exert some important benefits on the intestinal mucosa. Overall, based on the biological functions of the DEGs and the significantly enriched gene sets, we hypothesize that molecular pathways mostly impacted by YCW extract supplementation are those related to immunity. This suggests that yeast supplementation might play a key role in triggering anti-inflammatory mechanisms and an animal’s response to pathogens. Despite that, additional studies are needed to draw conclusive remarks about the molecular mechanisms modulated by YCW extract supplementation in this species (i.e. whole gene expression at different time points and/or in other target tissues).

## Conclusions

The dietary addition of yeast cell wall extracts improved broiler feed conversion. Moreover, despite the absence of any specific challenging issue, it improved gut conditions in terms of increased glycoconjugate secretion at the gut level, which would provide chickens with more defences against any pathogens. The reduction of the gut inflammatory status was associated with differences in the gut transcriptome, which highlights that YCW extracts might act in different ways, at the level of intestinal integrity or at different stages of the immune response. Based on these multiple effects and action mechanisms, the inclusion of yeast cell wall extracts can be considered useful to improve animal welfare and health under the challenging conditions of intensive rearing systems in broiler chickens.

## Supplementary information

**Additional file 1: Table S1.** Sequencing and mapping results. The table reports the RNA-Seq libraries sequenced including for each of them i) the number of raw reads obtained, ii) the number of reads after trimming and rRNAs removal iii) the number of mapped reads (and the percentage of mapped reads). **Table S2.** GSEA results. Enriched BIOCARTA Gene Sets (GS) at both the highest (up-regulation in “diet Y” condition) and lowest (up-regulation in “diet C” condition) part of the genes ranking are reported. *ES: enrichment score; NES: normalized enrichment score; NOM p-val: nominal P-value.*

## Data Availability

Raw Illumina sequencing data have been deposited in GenBank (SRA) under the SRA accessions SRR10358733-SRR10358740, and they will be published after manuscript acceptance. During reviewing process, BioProject’s metadata are available at the following link: https://dataview.ncbi.nlm.nih.gov/object/PRJNA579994?reviewer=8movh1n2dfhjipos7406hug71q The other datasets analysed in the current study are available from the corresponding author upon reasonable request.

## Abbreviations

BHLHE41: Basic helix-loop-helix family member E41
C: Control
CYP2C23B: Cytochrome P450, family 2, subfamily C, polypeptide 23b
DEG: Differentially expressed gene
EETs: Epoxyeicosatrienoic acids
IgG: Immunoglobulin G
GSEA: Gene set enrichment analysis
HUGO: Human Genome Organization
MOS: Mannan oligosaccharides
MSigDB: Molecular Signature Database
PAS: Periodic acid–Schiff
PBS: Phosphate buffer saline
PCR: Polymerase chain reaction
RIN: RNA integrity number
RMSE: Root mean square error
SEM: Standard error mean
STEAP4: Metalloreductase
TTC9: Tetratricopeptide repeat domain 9
Y: Yeast
YCW: Yeast cell wall
