# Supplementary material for: Effect of dietary supplementation with yeast cell wall extracts on performance and gut response in broiler chickens
Source: J Anim Sci Biotechnol. 2020 May 1;11:40. doi: 10.1186/s40104-020-00448-z (PMC7193382; doi:10.1186/s40104-020-00448-z)
Supplement: Supplementary file 1 — Additional file 1: Table S1. Sequencing and mapping results. The table reports the RNA-Seq libraries sequenced including for each of them i) the number of raw reads obtained, ii) the number of reads after trimming and rRNAs removal iii) the number of mapped reads (and the percentage of mapped reads). Table S2. GSEA results. Enriched BIOCARTA Gene Sets (GS) at both the highest (up-regulation in “diet Y” condition) and lowest (up-regulation in “diet C” condition) part of the genes ranking are reported. ES: enrichment score; NES: normalized enrichment score; NOM p-val: nominal P-value. [file 40104_2020_448_MOESM1_ESM.docx]

Supplementary Information for:

**Effect of the dietary supplementation with yeast cell wall extracts on performance and gut response in broiler chickens**

A. Pascual^1*^, M. Pauletto^1^, M. Giantin^1^, G. Radaelli^1^, C. Ballarin^1^, M. Birolo^2^, C. Zomeño^1^, M. Dacasto^1^, M. Vascellari^3^, M. Bortoletti^1^, G. Xiccato^2^, A. Trocino^1^

**Table S1.** **Sequencing and mapping results.** The table reports the RNA-seq libraries sequenced including for each of them i) the number of raw reads obtained, ii) the number of reads after trimming and rRNAs removal iii) the number of mapped reads (and the percentage of mapped reads).

| **Sample ID** | **N° raw reads** | **N° reads after trimming and rRNAs removal** | **N° of reads mapping (%)** |
| --- | --- | --- | --- |
| C_1 | 25,107,355 | 24,569,550 | 23,470,759 (95.5%) |
| C_2 | 28,696,711 | 28,175,126 | 26,961,542 (95.7%) |
| C_3 | 29,229,789 | 28,795,834 | 27,693,612 (96.2%) |
| C_4 | 24,872,313 | 15,824,707 | 15,255,606 (96.4%) |
| Y_1 | 21,440,507 | 21,069,758 | 20,248,235 (96.1%) |
| Y_2 | 25,940,693 | 25,567,068 | 24,567,534 (96.1%) |
| Y_3 | 21,620,563 | 21,250,169 | 20,395,808 (96.0%) |
| Y_4 | 17,993,873 | 10,066,664 | 9,651,228 (95.9%) |

**Table S2. GSEA results.** Enriched BIOCARTA Gene Sets (GS) at both the highest (up-regulation in “diet Y” condition) and lowest (up-regulation in “diet C” condition) part of the genes ranking are reported. *ES: enrichment score; NES: normalized enrichment score; NOM p-val: nominal P-value.*

| ***Enriched GS at the highest part of the genes ranking (i.e. genes up-regulated in “diet Y” group)*** | | | | | | |
| --- | --- | --- | --- | --- | --- | --- |
| **BIOCARTA GS** | **GS description** | **GS size** | **ES** | **NES** | **NOM *p-val*** | **Rank at max** |
| HES | Segmentation clock | 10 | 0.46 | 1.73 | 0.026 | 3699 |
| ***Enriched GS at the lowest part of the genes ranking (i.e. genes up-regulated in “diet C” group)*** | | | | | | |
| **BIOCARTA GS** | **GS brief description** | **GS size** | **ES** | **NES** | **NOM *p-val*** | **Rank at max** |
| RACCYCD | Influence of Ras and Rho proteins on G1 to S Transition | 22 | -0.42 | -2.44 | 0.000 | 5901 |
| RNA | Double Stranded RNA Induced Gene Expression | 9 | -0.64 | -2.36 | 0.000 | 1501 |
| KERATINOCYTE | Keratinocyte Differentiation | 37 | -0.31 | -2.22 | 0.002 | 5247 |
| NO2IL12 | NO2-dependent IL 12 Pathway in NK cells | 10 | -0.57 | -2.18 | 0.000 | 4756 |
| TCAPOPTOSIS | HIV Induced T Cell Apoptosis | 7 | -0.66 | -2.16 | 0.008 | 3696 |
| TCRA | Lck and Fyn tyrosine kinases in initiation of TCR Activation | 7 | -0.66 | -2.14 | 0.000 | 3696 |
| NFKB | NF-kB Signaling Pathway | 18 | -0.40 | -2.12 | 0.004 | 4136 |
| DEATH | Induction of apoptosis through DR3 and DR4/5 Death Receptors | 28 | -0.32 | -2.01 | 0.004 | 5936 |
| NTHI | NFkB activation by Nontypeable Hemophilus influenzae | 20 | -0.36 | -1.98 | 0.008 | 5378 |
| TOLL | Toll-Like Receptor Pathway | 22 | -0.35 | -1.97 | 0.006 | 4136 |
| TID | Chaperones modulate interferon Signaling Pathway | 15 | -0.42 | -1.95 | 0.008 | 5659 |
| RELA | Acetylation and Deacetylation of RelA in The Nucleus | 13 | -0.43 | -1.91 | 0.004 | 3701 |
| TCYTOTOXIC | T Cytotoxic Cell Surface Molecules | 8 | -0.55 | -1.90 | 0.012 | 830 |
| UCALPAIN | uCalpain and friends in Cell spread | 10 | -0.48 | -1.83 | 0.016 | 5725 |
| FAS | FAS signaling pathway ( CD95 ) | 29 | -0.28 | -1.82 | 0.013 | 5678 |
| HIVNEF | HIV-I Nef: negative effector of Fas and TNF | 51 | -0.21 | -1.80 | 0.020 | 5678 |
| FBW7 | Cyclin E Destruction Pathway | 9 | -0.49 | -1.80 | 0.014 | 5568 |
| IL1R | Signal transduction through IL1R | 23 | -0.30 | -1.76 | 0.024 | 4313 |
| TCR | T Cell Receptor Signaling Pathway | 35 | -0.26 | -1.76 | 0.011 | 6004 |
| CLASSIC | Classical Complement Pathway | 9 | -0.49 | -1.76 | 0.025 | 1959 |
| TSP1 | TSP-1 Induced Apoptosis in Microvascular Endothelial Cell | 7 | -0.54 | -1.76 | 0.023 | 5063 |
| IL2RB | IL-2 Receptor Beta Chain in T cell Activation | 30 | -0.26 | -1.76 | 0.023 | 5901 |
| CTL | CTL mediated immune response against target cells | 7 | -0.52 | -1.75 | 0.024 | 3696 |
| THELPER | T Helper Cell Surface Molecules | 7 | -0.52 | -1.73 | 0.020 | 3696 |
| IL6 | IL 6 signaling pathway | 16 | -0.36 | -1.72 | 0.024 | 2929 |
| 41BB | The 4-1BB-dependent immune response | 15 | -0.36 | -1.72 | 0.024 | 2670 |
| STRESS | TNF/Stress Related Signaling | 20 | -0.32 | -1.72 | 0.027 | 4136 |
| CTLA4 | Co-Stimulatory Signal During T-cell Activation | 14 | -0.38 | -1.71 | 0.016 | 3696 |
| LAIR | Cells and Molecules involved in local acute inflammatory response | 7 | -0.52 | -1.70 | 0.029 | 2091 |
| IL12 | IL12 and Stat4 Dependent Signaling Pathway in Th1 Development | 15 | -0.37 | -1.68 | 0.031 | 4756 |
| AKT | \|  \| AKT Signaling Pathway \| \| --- \| --- \| | 18 | -0.33 | -1.68 | 0.035 | 6165 |
| CD40 | CD40L Signaling Pathway | 14 | -0.37 | -1.68 | 0.023 | 5378 |
| ARAP | ADP-Ribosylation Factor | 14 | -0.37 | -1.66 | 0.035 | 3775 |
| RAS | Ras Signaling Pathway | 16 | -0.34 | -1.65 | 0.040 | 5901 |
| PML | Regulation of transcriptional activity by PML | 9 | -0.45 | -1.65 | 0.036 | 3589 |
| TH1TH2 | Th1/Th2 Differentiation | 10 | -0.42 | -1.63 | 0.041 | 5258 |
| TNFR2 | TNFR2 Signaling Pathway | 16 | -0.34 | -1.62 | 0.026 | 5936 |
| ACH | Role of nicotinic acetylcholine receptors in the regulation of apoptosis | 11 | -0.40 | -1.61 | 0.050 | 6620 |
| PLC | Phospholipase C Signaling Pathway | 8 | -0.46 | -1.59 | 0.049 | 5901 |
| CASPASE | Caspase Cascade in Apoptosis | 17 | -0.33 | -1.59 | 0.041 | 6773 |
| IL17 | IL 17 Signaling Pathway | 7 | -0.48 | -1.57 | 0.054 | 4143 |
